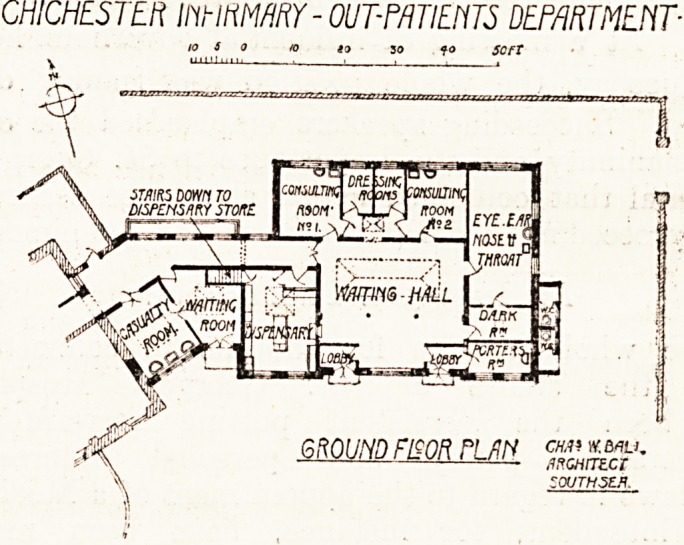# The Alterations at Chichester Infirmary

**Published:** 1914-01-24

**Authors:** 


### HOSPITAL ARCHITECTURE AND CONSTRUCTION.

### The Alterations at Chichester Infirmary.

We publish to-day further plans of the recent alterations at this infirmary.

The main building has had various additions on the north side, the most important of these being a new operation-room, surgeons'-room, and sterilising-room. These are all on the first-floor level. Below, on the ground floor, is a nurses' dining-room, with pantry adjoining, and the matron's office.

Two sanitary blocks have been built out; the western one provides a bath-room, w.c., and sink-room for the house surgeon on the ground floor, and a bath-room, sink-room, and w.c.s for the male wards on the first floor; the eastern block provides on the ground floor similar accommodation for the nurses and on the floor above for the female wards. The architect has been at great pains to provide cut-off lobbies between these sanitary blocks and the main building, notwithstanding the fact that a corridor intervenes between them and the wards, so that there is no question of direct access. These lobbies might very well have been dispensed with, and the distance between the wards and the offices materially lessened. A curious inconsistency is that the nurses' w.c. is on the corridor side of the cut-off lobby. If the lobby be a necessity at all, it is just as much required for the nurses' w.c. as for those for patients. Other alterations include the provision of testing-rooms, linen-rooms, and food cupboards in the ward kitchens.

The out-patient department forms a wing to the east end of the main block, and contains a waiting-hall with two entrances, two consulting-rooms, with two dressing-rooms on the north side, a large room for eye, ear, nose, and throat work with a dark-room attached, a dispensary, and a casualty-room with separate waiting-room. Presumably, one of the doors to the waiting-hall, close to which is the porter's-room, will be the entrance, and the other, which adjoins the dispensary, will be the exit. Two w.c.s outside the building form the sanitary provision for out-patients.

This somewhat crude arrangement might have been more carefully devised. Two w.c.s side by side seem hardly an ideal arrangement, and some provision ought to have been made for a urinal for male patients.

The architect is Mr. C. W. Ball, of Southsea.

## Figures and Tables

**Figure f1:**
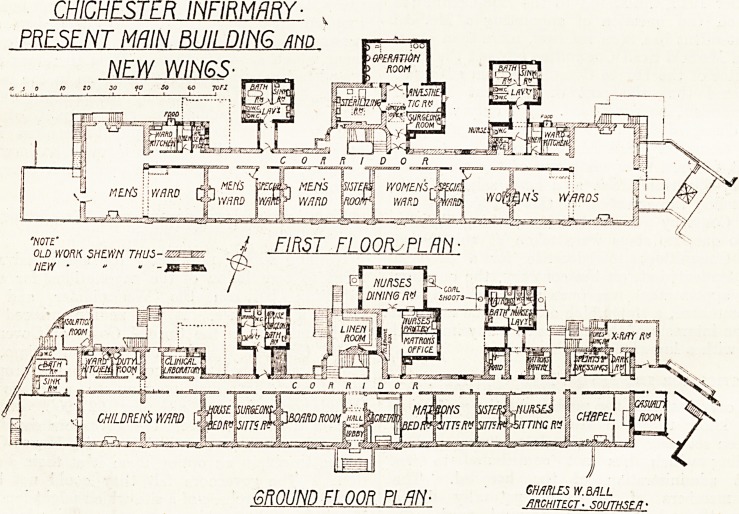


**Figure f2:**